# Laboratory diagnostics of chronic kidney disease in Serbia: Current practice and priorities for harmonisation

**DOI:** 10.5937/jomb0-65067

**Published:** 2026-06-16

**Authors:** Vera Lukić, Dušica Mrdaković, Neda Milinković

**Affiliations:** 1 Railway Healthcare Institute, Department of Laboratory Diagnostics, Belgrade; 2 University of Belgrade, Faculty of Pharmacy, Department of Medical Biochemistry, Belgrade; 3 Healthcare Centre Voždovac, Laboratory Department, Belgrade

**Keywords:** chronic kidney disease, estimated glomerular filtration rate, albumin-to-creatinine ratio, laboratory harmonisation, hronična bolest bubrega, procenjena jačina glomerulske filtracije, odnos albumin/kreatinin, laboratorijska harmonizacija

## Abstract

**Background:**

To assess current laboratory practices in chronic kidney disease (CKD) diagnostics in Serbia and identify areas requiring harmonisation in line with national and international guidelines.

**Methods:**

A cross-sectional, questionnaire-based survey was conducted between June and August 2025, with support from the Serbian Society of Medical Biochemists, which distributed invitations to its members working in medical laboratories across Serbia. Eighty-three laboratories participated (response rate 84.7%), representing primary, secondary, tertiary, and private healthcare sectors. Data were analysed descriptively.

## Abstract (sr)

**Uvod:**

Cilj ovog istraživanja bio je da se proceni aktuelna laboratorijska praksa u dijagnostici hronicne bolesti bubrega (HBB) u Republici Srbiji i identifikuju oblasti koje zahtevaju harmonizaciju u skladu sa nacionalnim i međunarodnim preporukama.

**Metode:**

Sprovedeno je presecno istraživanje zasnovano na upitniku u periodu jun-avgust 2025. godine, uz podršku Društva medicinskih biohemicara Srbije, koje je distribuiralo pozive svojim članovima zaposlenim u medicinsko-biohemijskim laboratorijama širom Srbije. U istraživanju su učestvovale 83 laboratorije (stopa odgovora 84,7%), koje su obuhvatile primarni, sekundarni, tercijarni i privatni sektor zdravstvene zaštite. Podaci su analizirani deskriptivno.

**Rezultati:**

Sve laboratorije određuju koncentraciju kreatinina u serumu, ali ostale ključne komponente dijagnostike HBB nisu dosledno implementirane. Procena jačine glomerulske filtracije (eGFR) zasnovana na kreatininu izračunava se u 63,9% laboratorija, dok se automatski prikazuje uz svaki rezultat kreatinina u 34,9%. Testiranje albuminurije dostupno je u 55,4% laboratorija; kvantitativno određivanje albumina sprovodi se u 31,3%, dok se odnos albumin/kreatinin (ACR) izračunava u 7,2% laboratorija. Određivanje cistatina C u serumu dostupno je u 7,2% laboratorija. U manje od polovine laboratorija (43,4%) istovremeno je dostupno i izračunavanje eGFR i testiranje albuminurije. Uočene su razlike u analitičkim metodama, referentnim intervalima, tipu uzorka urina i modelima izveštavanja. Integrisana primena eGFR i ACR najčešća je u privatnom sektoru, a najređa na nivou primarne zdravstvene zaštite.

**Zaključak:**

Laboratorijska dijagnostika HBB u Srbiji i dalje je dominantno zasnovana na kreatininu, uz ograničeno određivanje albuminurije i automatsko izveštavanje eGFR. Neophodne su šira primena ACR testa i standardizacija izveštavanja kako bi se omogućilo ranije otkrivanje i adekvatna procena rizika kod pacijenata sa HBB.

## Introduction

Chronic kidney disease (CKD) is widely recognised as a major public health problem, associated not only with progressive loss of kidney function but also with increased cardiovascular morbidity and mortality [1][2][3]. While global prevalence estimates range from 10% to 13% of adults, epidemiological data for Serbia remain limited. According to the Global Kidney Health Map, the estimated prevalence in Serbia is 12.9%, and recent population-based data have begun to emerge [4][5]. Available regional studies from the Balkans indicate systemic challenges, including delayed diagnosis, variability in laboratory testing practices, and restricted access to diagnostic resources, which collectively hinder effective CKD management [6][7].

Laboratory evaluation is central to CKD diagnosis and monitoring as KDIGO (Kidney Disease: Improving Global Outcomes) staging requires both eGFR and albuminuria assessment [8]. Serum creatinine remains the most widely used marker of kidney function; however, its levels are influenced by age, sex, and muscle mass, limiting diagnostic precision in certain populations. Cystatin C has emerged as a complementary marker that offers a more reliable assessment, particularly in elderly patients, children, and individuals with altered muscle mass. Urinary albumin, quantified as albumin-to-creatinine ratio (ACR) in spot urine samples, provides an early indicator of glomerular injury and is a strong prognostic marker for both renal and cardiovascular outcomes. International guidelines recommend the combined use of eGFR and albuminuria for CKD diagnosis, staging, and monitoring, emphasising the need for standardised assays, harmonised reference intervals, and consistent reporting practices [3][8].

Despite the publication of a national CKD guideline in Serbia [9], the extent to which laboratories adhere to these recommendations has not been systematically evaluated. Assessing current practice is a fundamental step in developing and refining evidence-based national strategies. The IFCC (International Federation of Clinical Chemistry and Laboratory Medicine) Committee on Renal Diseases has developed a standardised questionnaire translated into multiple languages to enable international benchmarking of laboratory practices [10]. The lack of such data for Serbia limits meaningful comparison with international standards. It may contribute to delayed CKD detection and suboptimal patient risk stratification.

Understanding the current laboratory landscape is particularly important within the Serbian healthcare system, which includes primary healthcare centres, secondary-level hospitals, tertiary-level (institutes, clinical hospital centres, and clinical centres), and private laboratories. These levels of care differ in available resources, technical capacity, and implementation of guidelines, potentially leading to heterogeneity in CKD diagnostics. Such heterogeneity may affect the comparability of results, early CKD detection, and appropriate patient risk stratification. International surveys have demonstrated that laboratory practices vary substantially even among high-income countries, and that factors such as guideline awareness, reagent availability, reimbursement policies, and laboratory information system integration strongly influence the quality, consistency, and clinical utility of CKD testing [11][12][13][14].

Therefore, the present study aimed to conduct a comprehensive survey of medical laboratories across Serbia to evaluate current CKD-related laboratory practices, assess heterogeneity in analytical methods, reference intervals, and testing volumes, and examine reporting models in relation to both national and international guideline recommendations. By doing so, the study seeks to provide a systematic overview of CKD diagnostics in the country and identify actionable areas for quality improvement.

## Materials and methods

This research was conducted as a cross-sectional, questionnaire-based study with the support of the Serbian Society of Medical Biochemists, which distributed the invitation to participate to its members working in medical laboratories across Serbia. The invitation explained the objectives and scope of the study. Participation was voluntary and anonymous. As the survey did not involve patient data, biological material, or identifiable institutional information, formal ethical approval was not required.

The survey was administered via Google Forms. Data were collected between June and August 2025. Each participating laboratory was asked to submit a single response reflecting itsroutine laboratory practice. The questionnaire was completed by medical or clinical biochemists responsible for laboratory operations. Responses were received from 83 laboratories.

A standardised questionnaire developed by the IFCC Committee on Renal Diseases served as the basis for the survey [10]. This instrument has been used previously in international initiatives to assess CKD laboratory practices. Only minor contextual adaptations were made (terminology and healthcare structure), while the core content and structure remained unchanged. The English version of the questionnaire is provided in Table 1. The questionnaire was divided into three sections, including questions or methodology, the number of daily requests, and the reporting of results for serum creatinine, cystatin C, and albuminuria.

**Table 1:** Questionaire used in the survey.

Questionnaire on Laboratory Diagnostics of Kidney Function in the Republic of Serbia	
Section 1. Serum Creatinine	
Question	Response options
Q1. Level of healthcare of the laboratory	Primary / Secondary / Tertiary / Private practice
Q2. Daily number of requests for serum creatinine measurement	<100 / 100-500 / >500
Q3. Method used for serum creatinine measurement	Enzymatic / Compensated Jaffe / Uncompensated Jaffe
Q4. Analyzer used for serum creatinine (manufacturer and model)	Open-ended response
Q5. Source of reference intervals for serum creatinine	Manufacturer's recommendation / Literature / Other
Q6. Are serum creatinine reference intervals sex-specific?	Yes / No
Q7. Sex-specific reference intervals (if applicable)	Open-ended response
Q8. Are serum creatinine reference intervals age-specific?	Yes / No
Q9. Age-specific reference intervals (if applicable)	Open-ended response
Q10. Do you calculate eGFR in individuals >18 years using serum creatinine?	Yes / No
Q11. eGFR formula used (adults)	MDRD / 2009 CKD-EPI creatinine / 2021 CKD-EPI creatinine / EKFC creatinine / Other
Q12. Do you calculate eGFR in individuals <18 years using serum creatinine?	Yes / No
Q13. Formula used for pediatric eGFR calculation	Open-ended response
Q14. Reporting of eGFR results	With every serum creatinine result / Only upon physician request
Q15. Format of eGFR reporting	Exact value for all results / Exact value only if <60 mL/ min/1.73 m[, otherwise reported as >60
Q16. Is CKD stage reported based on eGFR?	Yes / No
Section 2. Serum Cystatin C	
Q17. Do you measure serum cystatin C?	Yes / No
Q17a. Method for cystatin C measurement (if applicable)	Immunoturbidimetry / Immunonephelometry / Other
Q18. Analyzer used for cystatin C (manufacturer and model)	Open-ended response
Q19. Daily number of requests for cystatin C measurement	<20 / 20-100 / >100
Q20. Are cystatin C reference intervals sex-specific?	Yes / No
Q21. Sex-specific cystatin C reference intervals (if applicable)	Open-ended response
Q22. Are cystatin C reference intervals age-specific?	Yes / No
Q23. Age-specific cystatin C reference intervals (if applicable)	Open-ended response
Q24. Do you calculate eGFR based on cystatin C?	Yes / No
Q25. Formula used for cystatin C-based eGFR	2012 CKD-EPI cystatin C / EKFC cystatin C / Other
Q26. Is CKD stage reported based on cystatin C-derived eGFR?	Yes / No
Section 3. Urine Albumin	
Q27. Is urine albumin measured in your laboratory?	Yes / No
Q28. Type of urine albumin determination	Quantitative / Semi-quantitative / Qualitative
Q29. Daily number of requests for urine albumin measurement	<20 / 20-100 / >100
Q30. Method and analyzer used for urine albumin measurement	Open-ended response
Q31. Urine sample type for quantitative albumin measurement	24-hour urine / First morning sample / Random sample
Q32. Reporting format for single urine sample	Albumin concentration / Albumin-to-creatinine ratio (ACR) / Both
Q33. Are ACR reference intervals sex-specific?	Yes / No
Q34. Sex-specific ACR reference intervals (if applicable)	Open-ended response
Q35. Reference interval for albumin concentration in single urine sample	Open-ended response
Q36. Method for semi-quantitative/qualitative urine albumin testing	Open-ended response
Q37. Test strips used (name and manufacturer)	Open-ended response
Q38. Reporting format of dipstick albumin results	Numerical value / Positive-Negative
Q39. Reference interval for albumin in 24-hour urine	Open-ended response

Given the aim of the study to provide a comprehensive overview of current laboratory practice at the national level, a descriptive analytical approach was considered appropriate. Data were analysed descriptively using Excel (Microsoft Corporation, 2015). Frequencies and percentages were calculated for all categorical variables. Percentages were calculated using the number of laboratories responding to each question as the denominator; therefore, denominators may vary across variables due to missing responses.

## Results

### Characteristics of participating laboratories

The survey was completed by 83 laboratories (out of 98 invited; response rate: 84.7%), representing all levels of healthcare in Serbia, with primary care institutions constituting the largest group (Table 2).

**Table 2:** Characteristics of participating laboratories (n=83). Percentages were calculated using the total number of participating laboratories as the denominator (n = 83).

Healthcare level of <br>participating laboratories	Number of laboratories <br>n (%)
Primary	38 (45.8)
Secondary	23 (27.7)
Tertiary	14 (16.9)
Private laboratory practice	8 (9.6)

### Implementation of CKD diagnostic components

All participating laboratories measured serum creatinine. However, the implementation of additional CKD-relevant laboratory components was incomplete (Table 3). Test workload differed markedly between analytes: most laboratories reported 100-500 daily creatinine requests, whereas cystatin C and quantitative urinary albumin were requested in fewer than 20 samples per day in most laboratories performing these tests. This contrast illustrates the dominant role of creatinine as a routine marker compared with more advanced CKD biomarkers.

**Table 3:** Implementation of guideline-recommended CKD laboratory components in Serbia (n=83). Percentages were calculated using the total number of participating laboratories as the denominator (n=83).<br>CKD - chronic kidney disease; eGFR - estimated glomerular filtration rate; ACR - albumin-to-creatinine ratio.

CKD diagnostic component	Number of laboratories <br>n (%)
Serum creatinine measurement	83 (100)
Creatinine-based eGFR calculated (adults)	53 (63.9)
eGFR automatically reported with every creatinine result	29 (34.9)
eGFR reported only upon physician request	24 (28.9)
Albuminuria testing available (any method)	46 (55.4)
Quantitative urine albumin measurement	26 (31.3)
ACR reported in spot urine	6 (7.2)
Serum cystatin C measurement available	6 (7.2)
Cystatin C-based eGFR calculated	3 (3.6)
Both creatinine-based eGFR reporting and albuminuria testing	36 (43.4)

Creatinine-based eGFR reporting was not consistently implemented. Automatic reporting with every creatinine result was limited. Albuminuria testing was available in only a subset of laboratories; quantitative testing and ACR reporting in spot urine were accessible to only a minority. Serum cystatin C measurement and cystatin C-based eGFR calculation were uncommon. Detailed data are presented in Table 3.

### Serum creatinine measurement

Analytical practices related to serum creatinine showed considerable heterogeneity. Jaffe-based methods predominated, whereas enzymatic assays were less frequently used. Differences were also observed in the selection of reference intervals. Most laboratories (68/83, 81.9%) used manufacturer-recommended reference intervals, whereas 15 (18.1%) applied intervals from professional literature. Gender-specific reference intervals were widely used (71/83, 85.5%), whereas age-specific intervals were applied less consistently (41/83, 49.4%).

### Creatinine-based eGFR reporting practices

**Figure 1:**
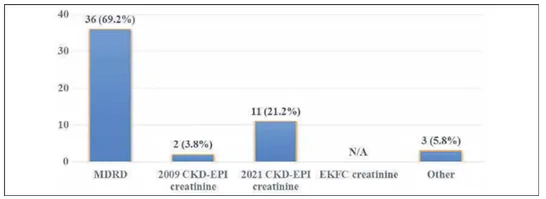
Prevalence of creatinine-based eGFR equations for adults used among laboratories in Serbia. Percentages are based on the 52 laboratories that answered the question on eGFR formulas (1 laboratory did not specify the equation used). eGFR - estimated glomerular filtration rate. MDRD - Modification of Diet in Renal Disease; CKD-EPI - Chronic Kidney Disease Epidemiology Consortium; EKFC - European Kidney Function Consortium

eGFR calculation in adults was not universally performed. Over one-third of participating laboratories did not calculate eGFR (Table 3). The distribution of equations used for adult eGFR calculation is presented in Figure 1.

Paediatric eGFR calculation was rare. Six laboratories specified the equation used in children: Schwartz (2/6), 2009 CKD-EPI (1/6), 2021 CKD-EPI (2/6), and Cockcroft-Gault (1/б). Notably, some laboratories reported using equations not validated for paediatric populations.

Reporting practices differed across laboratories. Some laboratories issued eGFR values with every creatinine result, whereas others provided eGFR only upon physician request. In addition, some laboratories reported all calculated eGFR values numerically, while others reported exact values only below 60 mL/min/1.73 m^2^ and expressed higher values as >60 mL/min/1.73 m^2^ (Table 3). Only one laboratory (1/53, 1.9%) reported an estimated stage of kidney insufficiency based on eGFR values.

### Serum cystatin C

Only a small number of laboratories (6/83) measured serum cystatin C, and just half of them calculated cystatin C-based eGFR. None of them reported CKD stage based on these results, nor did they apply age- or sex-specific reference intervals. Immunoturbidimetry was the predominant analytical method (5/6).

### Albuminuria testing

**Figure 2:**
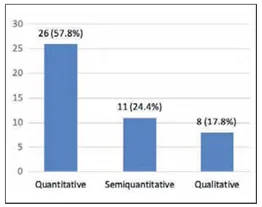
Variations of methods for urinary albumin determination. Percentages are based on the 45 laboratories that answered the question on albuminuria testing methods (1 laboratory did not specify the method used).

Urinary albumin testing was performed using quantitative, semi-quantitative, or qualitative approaches (Figure 2). Among laboratories performing quantitative measurements (n=26), immunoturbidimetry was the most commonly used analytical method.

**Figure 3:**
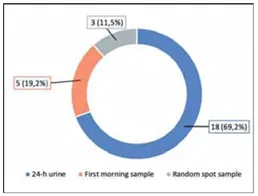
Prevalence of urine sample types for albumin determination. Percentages are based on the 26 laboratories performing quantitative urine albumin measurement.

Further heterogeneity was observed in the selection of urine samples for quantitative albumin determination. Twenty-four-hour urine collection was most frequently employed, followed by first-morning urine and random spot samples (Figure 3).

Laboratories also differed in how they expressed albuminuria results in spot urine, including reporting albumin concentration, ACR, or both (Table 4). Among laboratories reporting ACR (n = 6), the majority (83.3%) did not apply gender-specific reference intervals.

**Table 4:** Expression of albuminuria results among laboratories performing quantitative albumin testing in spot urine (n=21). Percentages were calculated using the number of laboratories that responded to the question on albuminuria result expression (n=21). Five laboratories did not specify the reporting format.<br>ACR - albumin-to-creatinine ratio.

Albuminuria result	Number of laboratories <br>n (%)
ACR	6 (28.6)
Albumin concentration	10 (47.6)
Both values	5 (23.8)

### Integration of CKD diagnostic components

**Figure 4:**
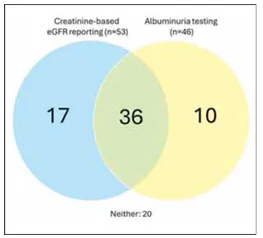
Integration of CKD diagnostic components in Serbian laboratories. CKD - chronic kidney disease

Less than half of laboratories performed both creatinine-based eGFR reporting and albuminuria testing (regardless of method, sample type, or result expression), indicating incomplete integration of the two principal laboratory components required for CKD detection. This overlap is shown in Figure 4. When considering the simultaneous implementation of eGFR reporting and quantitative albuminuria testing expressed as ACR, overall, 13% of participating laboratories offered it.

Implementation of key CKD diagnostic components varied substantially across different healthcare levels, as shown in Table 5. Tertiary institutions demonstrated greater integration of eGFR reporting and albuminuria testing than primary and secondary care laboratories. The highest rate of integration of CKD diagnostic components was achieved in private laboratories.

**Table 5:** Implementation of creatinine-based eGFR reporting and ACR testing according to healthcare level in Serbia (n=83). Percentages were calculated using the total number of laboratories within each healthcare level as the denominator. eGFR - estimated glomerular filtration rate; ACR - albumin-to-creatinine ratio.

Level of <br>healthcare	Number of laboratories			
	Total <br>laboratories <br>(n)	eGFR <br>reported <br>n (%)	ACR <br>testing <br>available <br>n (%)	Both eGFR <br>and ACR <br>implemented <br>n (%)
Primary	38	22 (57.9)	1 (2.6)	1 (2.6)
Secondary	23	13 (56.5)	4 (17.4)	3 (13.0)
Tertiary	14	10 (71.4)	2 (14.3)	2 (14.3)
Private<br>laboratories	8	8 (100)	4 (50.0)	4 (50.0)

## Discussion

This survey provides a structured overview of CKD-related laboratory practices in Serbia, offering insight into the real-world implementation of guideline-recommended diagnostic components across different levels of healthcare. The findings delineate how CKD laboratory diagnostics are currently implemented, including biomarker availability, analytical approaches, reference intervals, and reporting models. Across nearly all examined domains, substantial heterogeneity was observed, indicating that current practice is only partially aligned with national and international recommendations [8][9][13].

Serum creatinine was universally measured, confirming its role as the foundational biochemical marker of kidney function. This is consistent with international experience and reflects the long-standing reliance on creatinine as the entry point to laboratory CKD assessment [13][15]. However, pronounced variability was observed in analytical methodology and interpretation. The predominance of Jaffe-based methods, which are more susceptible to analytical interference and bias, may be clinically relevant, particularly at lower creatinine concentrations. Such variability is especially important in children, older adults, and individuals with reduced muscle mass, where small analytical errors may influence kidney function classification. The inconsistent application of age- and sex-specific reference intervals further contributes to interlaboratory variability. Although the type of creatinine assay was recorded, the survey did not specifically assess calibration traceability to IDMS reference methods. Enzymatic and compensated Jaffe assays are commonly aligned with international reference standards, but method type alone does not guarantee standardisation. As current eGFR equations assume IDMS-standardised creatinine, this represents an important aspect of analytical harmonisation that warrants further evaluation in the national laboratory setting. Similar challenges in creatinine standardisation and reference interval harmonisation have been described in European and global laboratory surveys [13][14].

The heterogeneity observed in creatinine measurement extends into eGFR reporting. Although eGFR calculation has become an integral component of modern CKD diagnostics, its implementation in Serbia is not universal. It should be noted that 39.1% of laboratories do not perform eGFR calculation at all. A substantial proportion of laboratories (65.1%) do not automatically report eGFR with every creatinine result, leaving clinicians to receive isolated creatinine values without contextual interpretation of kidney function. These data suggest that the implementation of eGFR reporting in Serbia has not yet reached the level of European countries [13]. In clinical practice, the absence of automatic eGFR reporting may lead to underrecognition of reduced kidney function, delayed diagnosis of CKD, and missed opportunities for timely intervention and referral. Automatic eGFR reporting is a pivotal system-level feature, as it ensures that kidney function assessment accompanies every creatinine measurement without requiring additional clinical requests. International experience indicates that automatic eGFR reporting is associated with improved recognition and staging of CKD [12][16]. Variability was also observed in the expression of eGFR values, including reporting exact numerical results versus threshold-based formats. However, guidelines recommend reporting values above 60 mL/min/1.73 m^2^ as »>60 mL/min/1.73 m^2^« [8]. Such variability in reporting models has been noted in other regions and represents a persistent challenge to harmonisation [12][13].

Paediatric eGFR reporting was uncommon. Although the survey did not determine whether all participating laboratories routinely serve paediatric populations, the finding suggests that paediatric-specific equations are not widely embedded in laboratory reporting systems. Accurate assessment of kidney function in children requires age-appropriate equations, given developmental changes in muscle mass and creatinine production [8]. Similar gaps in paediatric eGFR implementation have been reported in European surveys [13].

Cystatin C, recommended as a complementary marker of kidney function when creatinine-based estimates are less reliable [8], was available in only a small fraction of laboratories. Limited use of cystatin C testing has also been reported in other middle-income settings [13][14]. Even where cystatin C was measured, eGFR calculation based on cystatin C was not universal, and clinical interpretation remained limited. These findings indicate that the potential advantages of combined creatinine-cystatin C equations are not yet widely realised in routine practice.

Testing volumes reported for CKD-related biomarkers provide additional insight. The low daily numbers of cystatin C and albuminuria measurements suggest that these tests, even where technically available, may not be fully integrated into routine clinical pathways. Test utilisation patterns may reflect a combination of analytical capacity, clinical awareness, referral practices, and reimbursement structures. Although these determinants were not directly assessed in the present study, limited use of albuminuria and cystatin C testing may contribute to underrecognition of early CKD stages.

Albuminuria testing, a key component of KDIGO-based CKD classification and risk stratification [8], was not universally available. The limited availability of ACR indicates that albuminuria assessment remains the most underdeveloped component of CKD laboratory diagnostics in Serbia, despite its central role in KDIGO-based risk stratification [8]. Considerable heterogeneity was observed in sample types, analytical methods, and reporting formats. Laboratories differed in their use of 24-hour urine collections, first-morning samples, or random spot samples, and results were reported as albumin concentration, ACR, or both. The frequent use of 24-hour urine collections rather than spot ACR suggests that diagnostic workflows are still influenced by older practices rather than by current guideline-endorsed strategies. The observed ACR implementation rate is lower than that reported in recent European laboratory surveys, where spot ACR testing is increasingly established as routine practice [13]. Limited use of sex-specific ACR reference intervals further reduces comparability. International surveys have similarly identified albuminuria testing as an area with persistent variability and insufficient standardisation [12][13][15]. However, the extent of variability observed in the present study suggests that challenges in harmonisation may be more pronounced in settings with heterogeneous healthcare structures. Because KDIGO classification relies on both eGFR and albuminuria, incomplete implementation of either component may limit the full application of risk stratification frameworks. Reimbursement policies and test pricing may represent additional barriers, although these aspects were not assessed.

A key finding of this survey is the limited integration of the two principal laboratory components required for CKD detection - eGFR reporting and albuminuria assessment. Although both tests are available in parts of the system individually, their combined implementation remains inconsistent. The limited co-implementation of automatic eGFR reporting and albuminuria testing represents the central structural gap in current CKD laboratory practice. CKD laboratory diagnostics in Serbia remain predominantly creatinine-based, with insufficient integration of albuminuria. As KDIGO classification requires the combined interpretation of eGFR and albuminuria [8], incomplete implementation of either component may result in inadequate CKD staging and suboptimal risk stratification. This pattern suggests that institutional resources, local practices, and the degree of guideline implementation may influence the completeness of CKD diagnostics. Differences across healthcare levels, including private laboratories, indicate potential areas for improvement in CKD diagnostic practices.

Several structural and organisational factors may contribute to this situation. Differences in laboratory information system configurations, resource availability, local practice traditions, and interpretation of existing recommendations may all influence implementation. These factors were not directly examined in the present study and, therefore, should be considered potential rather than proven determinants. Comparable gaps between guideline recommendations and real-world laboratory practice have been described internationally [11][16][17]. Experiences from other countries indicate that national surveys followed by professional consensus recommendations, education programs, and periodic re-evaluation can lead to measurable improvements in CKD laboratory practice [16][17][18]. Harmonisation of CKD laboratory diagnostics in Serbia is therefore less a matter of analytical capability than of coordinated implementation, reporting policy, and integration of laboratory information systems. Several interventions, particularly broader implementation of automatic eGFR reporting and increased use of ACR in spot urine, primarily require organisational coordination and LIS optimisation rather than major technological investments. A particularly important structural gap is the limited availability of urine albumin testing at the primary healthcare level, where early CKD detection should ideally take place, potentially reflecting reimbursement policies that do not support routine implementation at this level of care.

The clinical implications of observed heterogeneity are relevant. Variability in laboratory practice may influence CKD detection, staging, and risk stratification, thereby affecting cardiovascular risk assessment, referral pathways, and long-term management. From a public health perspective, lack of standardised laboratory data may also limit the development of consistent national CKD surveillance systems [3][19].

This study has limitations. Data were based on self-reporting and may not fully reflect actual analytical performance or LIS configurations. Participation was voluntary, and the survey did not constitute a complete national census; therefore, results reflect practices among participating laboratories and may not be fully representative of all laboratories in Serbia. The study design was descriptive and did not assess causal relationships or clinical outcomes. The survey did not directly assess the calibration traceability of creatinine assays to isotope-dilution mass spectrometry (IDMS) reference methods. Participation bias toward more organised or guideline-aware laboratories cannot be excluded. These limitations are inherent to survey-based studies; however, they do not diminish the value of the findings as a baseline assessment of current laboratory practice. Despite the limitations, the high response rate and inclusion of laboratories across all levels of healthcare provide a robust overview of current practice and a valuable basis for future national and international comparisons.

## Conclusions

CKD-related laboratory practice in Serbia is characterised by substantial heterogeneity and only partial alignment with international recommendations. While serum creatinine is universally measured, analytical methods and reference interval use are not fully harmonised. Automatic eGFR reporting is not universal, albuminuria testing (particularly ACR in spot urine) is incompletely implemented, and cystatin C remains rarely available. Only a minority of laboratories simultaneously implement both eGFR reporting and albuminuria testing required for KDIGO-based CKD staging.

The findings identify specific system-level priorities for harmonisation, particularly wider implementation of automatic eGFR reporting, expansion of ACR testing in spot urine, and improved integration of CKD diagnostic components. Together with continued professional education and improved LIS integration, such measures would strengthen early CKD detection, staging, and risk stratification, bringing laboratory practice in Serbia closer to international standards and supporting improved patient care.

## Dodatak

### Acknowledgements

The authors gratefully acknowledge Assistant Professor Dr Snežana Jovičić, President of the Society of Medical Biochemists of Serbia, for her support in distributing the survey.

### Authors' contribution

Vera Lukić and Dušica Mrdaković share first authorship.

### Conflict of interest statement

All the authors declare that they have no conflict of interest in this work.
